# Supplementary material for: Circulating neutrophil extracellular trap (NET)-forming ‘rogue’ neutrophil subset, immunotype [DEspR + CD11b +], mediate multi-organ failure in COVID-19—an observational study
Source: Transl Med Commun. 2023 Apr 18;8(1):12. doi: 10.1186/s41231-023-00143-x (PMC10111078; doi:10.1186/s41231-023-00143-x)
Supplement: Supplementary file 1 — Additional file 1: Supplemental Methods. Figure S1. Scatter plots and Spearman correlation coefficients of DEspR+[NET+N] counts (103or K/μL)with measures of severity in COVID-19 at timepoint-1 (t1) and timepoint-2 (t2). [file 41231_2023_143_MOESM1_ESM.pdf]

Supplemental Methods.

Rigor and Reproducibility.
Rigor was attained in all aspects; all recruited subjects were included in the study. Operators in each aspect of the study were blinded to the information/results of the other components of the study. Experimental confounders were minimized by the use of identical protocols for blood cytology slide preparation shortly after blood draw – within 1 hr at BUSM in EDTA-anticoagulated blood, and within 1-3 hrs at MMC in ACD anticoagulated blood, and by single site immunofluorescence cytology followed by independent 3rd party automated confocal quantitative analysis of DEspR±CD11b±[NET+Ns] using fluorescence and shape analysis algorithms.

All measurements were obtained following protocols that minimize experimental variables. We measured subset-specific NET+N levels directly in the circulation focusing on immunotyped DEspR+CD11b+ NET+N levels (mediator) without prior neutrophil isolation. Cytology slides were prepared within 1-hour (BMC) or within 1-3 hours (MMC).] We obtained the peak SOFA-score without neurological component measure, Glasgow Coma Scale, in order to eliminate variability and logistic difficulties in assessing the neurological component in critically ill sedated patients.[1],[2] We used a second clinically relevant outcome score representing mortality and severity that require longer durations of ICU-care, ICU-free days at day 28 with competing risk of death scored as -1, ICUFD.

We validated the method to measure intravascular levels of DEspR±CD11b± NET±Ns on immunostained cytology slides via automated confocal microscopy and shape-analysis for circularity index to define NET+Ns expressing both DEspR+ survival receptor and CD11b+ activation marker.[3] As neutrophils continue to react *ex vivo*, cytology slides are prepared using identical protocols within a pre-specified time from blood draw to eliminate experimental variables in measurements.[4]

Inclusion and exclusion criteria.
Inclusion criteria comprised of documented COVID-19 diagnosis by PCR with symptomatic respiratory distress requiring admission to the hospital or ICU, age > 18 years, informed consent for blood and clinical data collection. Exclusion criteria included pregnant females, prisoners. At BUSM, subject recruitment focused on critically ill COVID-19 patients in the ICU on ventilator; at MMC, subject recruitment focused on moderate-severe COVID-19 with respiratory distress in the ICU or step-down unit.

Data Collection.
Timepoints for blood sample collection were: t1: shortly after admission to the hospital (~1 day) or ICU (average ~5.5-days due to increased time needed to get informed consent from legal representative during COVID-19 restriction period, and t2: day before hospital/ICU discharge or death. Sequential Organ Failure Assessment (SOFA)-scores were obtained without the neurological component. ARDS diagnosis was defined using the Berlin definition. The SpO2/FiO2 was used when PaO2 was not available, and converted to SaO2/FiO2 for the SF-ratio. CBC-differential was used to obtain the absolute neutrophil count, absolute lymphocyte count, and neutrophil-to-lymphocyte ratio (NLR). Clinical data and laboratory measurements (BUSM and MMC) were collated by one co-author distinct from co-authors performing causal mediation analysis.

Blood collection

Peripheral blood patient samples were collected into K2-EDTA vacutainer tubes (FisherScientific, MA) at BMC and in ACD tubes at MMC. Blood smears were prepared within 1-hour from blood draw at BUSM, and within 1-3 hours at MMC following institutional approved physical containment and BSL2 precautions in handling of potentially contaminated human blood samples. Whole blood cytology slides or "blood smears" were prepared on PLUS slides (FisherScientific), then fixed in -20 °C methanol x 10 minutes, then stored dry in -20 °C freezer. Cold methanol protects cell morphology, protein, and nucleic acid integrity and number,[5] and disinfects specimens for infectious SARS CoV-2 and other viruses.

Immunofluorescence staining of NET-forming neutrophils [NET+Ns]

Blood smears were prepared by capillary action from EDTA anticoagulated whole blood (10 µL) samples on a Superfrost Plus Microscope slide (Fisher Scientific, cat# 12-550-15). Blood smears were air dried for 1-hour then fixed with pre-chilled 100% Methanol at -20 °C for 10 min; fixed slides were stored dry in -20 °C freezer for future immunostaining. Immunofluorescence (IF)-staining to detect NET-forming neutrophils was done as described[6]. We used anti-DEspR hu6g8-AF568, anti-CD11b-AF488, and anti-citH3-AF488, or anti-Histone H3 [Citrulline Arg17, Citrulline Arg8, Citrulline Arg2) antibody (Novus Biologicals NB100-57135).

Humanized DEspR IgG4[S228P] antibody (in house stock) was labeled with AF568, citH3 antibody was labeled with AF488; conjugated CD11b-AF488 was purchased (BioLegend 101217, M1/70 clone, rat IgG2b. AF488 and AF568 Fluorophore labeling kits were used following manufacturer's protocol (ThermoFisher Scientific, Invitrogen A20184 [AF568], A20181 [AF488]). 1X phosphate buffer saline (PBS) with heat-inactivated, tissue culture grade 5% fetal bovine serum (FBS) was used in washes, blocking and binding steps.

Semi-automated Quantitation of [NET+Ns] subsets

Nikon Imaging Laboratory (Cambridge MA) performed quantitative imaging analysis blinded to patient information. Slides were imaged with a Nikon Ti2-E Widefield microscope equipped with a Plan Apo λ 20x objective and Spectra LED light source and controlled by NIS-Elements. Briefly, an automated, JOBS routine in NIS-Elements was used to image 100 evenly spaced positions along an entire slide. At each position, focus was automatically adjusted with the Perfect Focus System (PFS) and then sequential images with the 395 (Blue), 470 (Green) and 555 (Red) nm LED light sources to detect DAPI (nuclei), Alexa Fluor 488 (CD11b) and Alexa Fluor 568 (DEspR, hu6g8), respectively. Each stack of 100 images was then processed with a General Analysis 3 algorithm in NIS-Elements to segment the nuclei, measure their circularity (Circularity = 4π [area/perimeter^2], area of minimum circle enclosing NET-forming neutrophil, perimeter of NET-forming neutrophil with all DNA-extrusions], and quantify the signal intensity of any co-localized CD11b and DEspR expression. Data were exported to a CSV file where the final scoring is completed in Excel.

---

[1] Kashyap R, Sherani KM, Dutt T, Gnanapandithan K, Sagar M, Vallabhajosyula S, Vakil AP, Surani S. Current Utility of Sequential Organ Failure Assessment Score: A Literature Review and Future Directions. Open Respir Med J. 2021 Apr 13;15:1-6. doi: 10.2174/1874306402115010001. PMID: 34249175; PMCID: PMC8227444.

[2] Nates JL, Cárdenas-Turanzas M, Wakefield C, Kish Wallace S, Shaw A, Samuels JA, Ensor J, Price KJ. Automating and simplifying the SOFA score in critically ill patients with cancer. Health Informatics J. 2010 Mar;16(1):35-47. doi: 10.1177/1460458209353558. PMID: 20413411.

[3] Herrera VLM, Walkey AJ, Nguyen MQ, Gromisch CM, Mosaddhegi JZ, Gromisch MS, Jundi B, Lukassen S, Carstensen S, Denis R, Belkina AC, Baron RM, Pinilla-Vera M, Mueller M, Kimberly WT, Goldstein JN, Lehmann I, Shih AR, Eils R, Levy BD, Ruiz-Opazo N. A targetable 'rogue' neutrophil-subset, [CD11b+DEspR+] immunotype, is associated with severity and mortality in acute respiratory distress syndrome (ARDS) and COVID-19-ARDS. Sci Rep. 2022 Apr 4;12(1):5583. doi: 10.1038/s41598-022-09343-1. PMID: 35379853; PMCID: PMC8977568.

[4] *ibid.* Herrera VLM, et al, 2022.

[5] Alles J, Karaiskos N, Praktiknjo SD, Grosswendt S, Wahle P, Ruffault PL, Ayoub S, Schreyer L, Boltengagen A, Birchmeier C, Zinzen R, Kocks C, Rajewsky N. Cell fixation and preservation for droplet-based single-cell transcriptomics. BMC Biol. 2017 May 19;15(1):44. doi: 10.1186/s12915-017-0383-5. PMID: 28526029; PMCID: PMC5438562.

[6] *Loc cit.* Herrera VLM, et al, 2022.

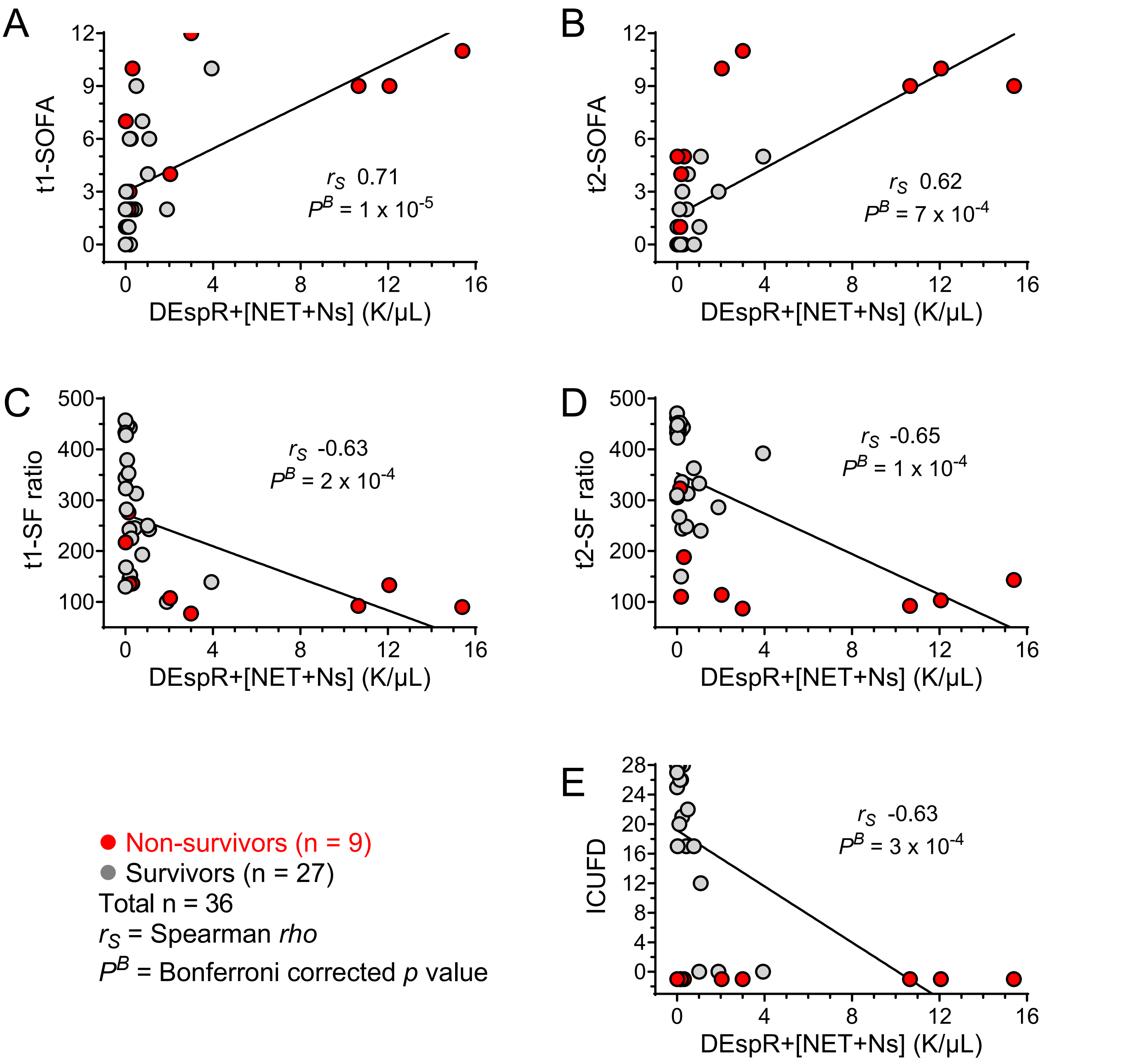

Figure S1. Scatter plots and Spearman correlation coefficients of DEspR+[NET+N] counts (10^3 or K/μL) with measures of severity in COVID-19 at timepoint-1 (t1) and timepoint-2 (t2). NET+Ns linear relationship with: **(A)** t1-SOFA score (non-neurological SOFA), **(B)** t2-SOFA score, **(C)** t1-SF ratio, and **(D)** t2-SF ratio; **(E)** ICUFD (ICUFD at day 28, with competing risk of death -1). Red ●, COVID-19 patient died in the ICU; grey ●, COVID-19 discharged from the ICU/hospital. Spearman correlation coefficient rho r_S and corresponding Bonferroni corrected *P*-values (*P* value x 7) for 7 hypotheses tested in Table 2.
